# Cortical activity associated with the detection of temporal gaps in tones: a magnetoencephalography study

**DOI:** 10.3389/fnhum.2014.00763

**Published:** 2014-10-09

**Authors:** Takako Mitsudo, Naruhito Hironaga, Shuji Mori

**Affiliations:** ^1^Department of Informatics, Faculty of Information Science and Electrical Engineering, Kyushu UniversityFukuoka, Japan; ^2^Department of Clinical Neurophysiology, Neurological Institute, Faculty of Medicine, Graduate School of Medical Sciences, Kyushu UniversityFukuoka, Japan

**Keywords:** gap detection, within-frequency (WF), between-frequency (BF), regional activity (RA), cortical tonotopy

## Abstract

We used magnetoencephalogram (MEG) in two experiments to investigate spatio-temporal profiles of brain responses to gaps in tones. Stimuli consisted of leading and trailing markers with gaps between the two markers of 0, 30, or 80 ms. Leading and trailing markers were 300 ms pure tones at 800 or 3200 Hz.Two conditions were examined: the within-frequency (WF) condition in which the leading and trailing markers had identical frequencies, and the between-frequency (BF) condition in which they had different frequencies. Using minimum norm estimates (MNE), we localized the source activations at the time of the peak response to the trailing markers. Results showed that MEG signals in response to 800 and 3200 Hz tones were localized in different regions within the auditory cortex, indicating that the frequency pathways activated by the two markers were spatially represented.The time course of regional activity (RA) was extracted from each localized region for each condition. In Experiment 1, which used a continuous tone for the WF 0-ms stimulus, the N1m amplitude for the trailing marker in the WF condition differed depending on gap duration but not tonal frequency. In contrast, N1m amplitude in BF conditions differed depending on the frequency of the trailing marker. In Experiment 2, in which the 0-ms gap stimulus in the WF condition was made from two markers and included an amplitude reduction in the middle, the amplitude in WF and BF conditions changed depending on frequency, but not gap duration.The difference in temporal characteristics betweenWF and BF conditions could be observed in the RA.

## INTRODUCTION

The human auditory system is sensitive to temporal changes in sounds. Gap detection is a frequently used task that measures auditory temporal resolution by requiring a listener to judge whether a stimulus contains a brief silent interval (gap). When leading and trailing markers share the same frequency, this task is referred to as a within-frequency (WF) detection task (Formby and Forrest, 1991; Formby et al., 1998; Phillips, 1999), and the gap-detection threshold (i.e., the minimally detectable gap duration) is usually found to be around 2–3 ms (Plomp, 1964; Penner, 1977). When the leading and trailing markers differ in frequency, the task is referred to as a between-frequency (BF) detection task. Psychophysical evidence has shown that gap detection becomes more difficult as the frequency difference between the leading and trailing markers increases; the gap-detection threshold can be as high as 50 ms when the frequencies are separated by two octaves (Formby and Forrest, 1991; Phillips et al., 1997; Formby et al., 1998; Phillips, 1999).

In contrast to the many psychophysical studies concerning WF-gap detection and differences between WF and BF gap-detection thresholds (Moore et al., 1989; Phillips et al., 1997; Phillips, 1999; Heinrich and Schneider, 2006), physiological studies regarding BF conditions are relatively few and the underlying neural mechanisms are not yet well understood. Electrophysiological studies that have investigated cortical responses to BF- and WF-gap detection have highlighted the importance of trailing-marker onset in relation to leading marker offset (Eggermont, 2000; Lister et al., 2007; Ross et al., 2010). Lister et al. (2007) recorded electroencephalograms (EEG) containing P1-N1-P2 auditory evoked responses to leading and training markers in WF and BF conditions. In the BF condition, trailing-marker onset elicited P1-N1-P2 responses for all gap durations, while in the WF condition they did so only when gaps were at least as long as the gap-detection threshold. Heinrich et al. (2004) focused on central processing in BF-gap detection by recording mismatch negativity (MMN) waves in an odd-ball paradigm. The results showed no significant effect of gap duration on MMN amplitude and suggested that primary auditory cortex plays a central role in the computation required for WF- and BF-gap detection.

To further investigate activity in the auditory cortex in response to silent gaps under BF conditions, we recorded magnetoencephalograms (MEG), a technique not yet used in studies of BF-gap detection. Specifically, we measured auditory evoked fields (AEFs) to reveal the spatio-temporal characteristics of cortical activity that may underlie psychophysical performance in WF- and BF-gap detection. MEG was conducted with minimum norm estimate (MNE), a visualization method that uses distributed source modeling with additional *a priori* constraints and can represent a number of local or distributed sources (Hamalainen and Ilmoniemi, 1994). Owing to high temporal and spatial resolution, MEG-source analysis can extract fine temporal information from localized regions. As in EEG studies that showed clear differences between WF and BF conditions in response to the trailing marker (Lister et al., 2007), here we observed the response to trailing-marker onsets in concentrated regions and looked in the auditory cortex for activity related to the gaps.

We examined spatial characteristics of cortical activity in terms of the frequency pathways for leading and trailing markers that were represented by tonotopic organization of auditory cortex. Neurons responding best to tones at specific frequencies are known to form tonotopic maps in auditory cortex (Woolsey, 1960). Studies using functional magnetic resonance imaging (fMRI) and MEG have shown that tonotopic organization exists not only in non-human primates but also in the human auditory cortex (e.g., Pantev et al., 1988, 1995; Formisano et al., 2003). In the present study, we used MNE and the marked inspection region of interest (iROI) to localize source activations at the time of the peak response to the trailing markers. We then analyzed the regional activities (RAs) in the iROI to compare the time courses across conditions. By visualizing activity in the auditory cortex during both BF and WF conditions, we were able to observe how the leading and trailing markers of different frequencies activated distinct areas in the auditory cortex.

The present study consisted of two experiments which differed primarily in the construction of the 0-ms-gap stimulus in the WF condition. In Experiment 1, it was a pure tone lasting 600-ms, which matched the total length of leading and trailing markers used in other conditions. In Experiment 2, it was constructed from two pure tones, each lasting 300 ms. While amplitude was not reduced in the middle of the 0-ms-gap stimulus in Experiment 1, it was reduced between the two markers in Experiment2. Thus, in Experiment 2, the 0-ms-gap stimulus was qualitatively similar to the other stimuli, while in Experiment 1 it was slightly different.

## MATERIALS AND METHODS

### PARTICIPANTS

Ten (five females, aged 23–53years) and six (four females, aged 23–37years) healthy volunteers participated in Experiment 1 and 2, respectively. No participants reported a hearing deficit or had difficulty hearing any of the stimuli used in the experiment. Informed consent was obtained from each participant after receiving an explanation of the purpose and procedures of the experiment. The study was approved by the Kyushu University Ethics Committee of the Faculty of Information Science and Electrical Engineering.

### STIMULI AND PROCEDURE

Stimuli were synthesized on a personal computer (Dimension 4500C, DELL Inc., Round Rock, TX, USA) with a sampling frequency of 44.1kHz. Stimuli were presented by a personal computer using STIM2 software (Neuroscan Co. Ltd., Charlotte, NC, USA), were amplified (PS3001, DMglobal Co. Ltd., Mahwah, NJ, USA), and presented monaurally to the participants’ right ears via a pair of inserted earphones (ER-3A, Etymotic Research Inc., Elk Grove Village, IL, USA). All stimuli were presented at 82dB SPL measured by a sound-level meter with a 1/2-inch condenser microphone (Brüel and Kjær, models 2250 and 4192). Participants were instructed to listen passively to the stimuli, stay alert, and keep their eyes open throughout each experimental block. Each participant’s behavior during MEG measurement was monitored using a TV-monitor system, and auditory responses were checked using online averaging.

#### Experiment 1

**FIGURE 1:**
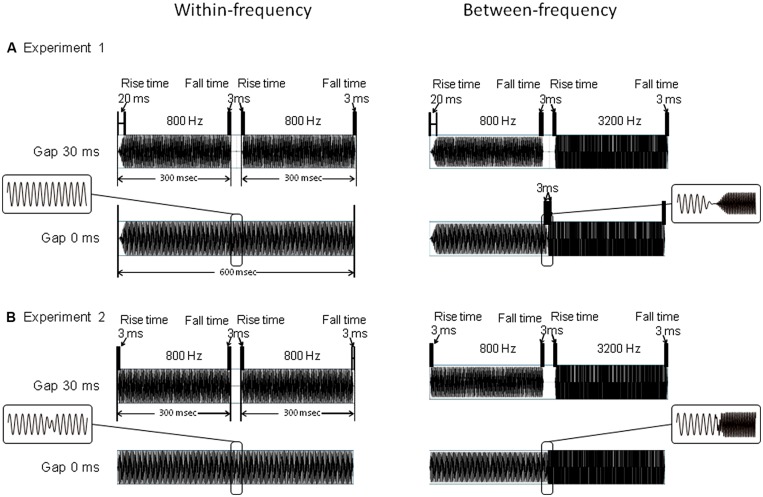
**Stimulus constructions of Experiment 1 **(A)** and Experiment 2 (B).** For both Experiments 1 and 2, upper figures represent the gap 30-ms stimuli, while lower figures represent the gap 0-ms stimuli. Figures on the left represent WF conditions of 800/800 Hz, and figures on the right represent BF conditions of 800/3200 Hz. In Experiment 1, leading marker includes 20-ms rise times and 3-ms fall times, while the trailing marker contains a 3-ms rise/fall time. In Experiment 2, leading and the trailing markers both contain a rise/fall time of 3-ms. For 30-ms gap stimulus, 300-ms markers are separated by a 30-ms gap in both Experiments 1 and 2. In Experiment 2, for 0-ms gap stimulus, two markers were overlapped to such an extent that the starting point of the rise time of the trailing marker and the starting point of the fall time of the leading marker were temporally aligned at the same position. The ranges ∼10 ms before and after the gaps were enlarged and displayed in flames.

Except for the WF 0-ms-gap stimulus, all stimuli consisted of leading and trailing markers, which were pure tones lasting 300 ms each. The 300-ms leading marker included 20-ms rise and 3-ms fall times, and the trailing marker contained 3-ms rise and 3-ms fall times (**Figure 1A**). For the WF condition, the frequencies of the two markers were identical to each other, being either 800/800 or 3200/3200 Hz. For the BF condition, the frequencies of the two markers were different, being either 800/3200 or 3200/800 Hz. The gap duration was either 0 (no gap), 30, or 80 ms. The 30- and 80-ms-gap durations were used to match those found in the gap-detection literature (Phillips et al., 1997; Elangovan and Stuart, 2008), which show that while both durations are clearly detectable in WF conditions, in BF conditions, the 30-ms gap is close to gap-detection threshold while the 80-ms gap is well beyond threshold. In the WF condition, the 0-ms-gap stimulus was a pure tone lasting 600 ms, with no amplitude reduction in the middle (**Figure 1A**, left). In the BF condition, it was a concatenation of leading and trailing markers (both 300 ms). This resulted in amplitude reduction in the middle owing to their 3-ms rise and fall times (**Figure 1A**, right). For each frequency combination (FC), each gap-duration stimulus was presented 80 times in pseudo-random order. These 960 trials (4 FCs × 3 gap durations × 80 trials) were divided into four blocks of 240 trials. Inter-trial intervals randomly varied from 1.5 to 1.8 s. Condition order was counterbalanced across participants.

#### Experiment 2

The stimuli were identical to those of Experiment 1, except in two respects. First, both the leading and the trailing markers contained 3-ms rise/fall times. Second, the 0-ms-gap stimuli for both conditions consisted of leading and trailing markers, with the fall time of the leading markers and the rise time of the trailing markers overlapping each other (**Figure 1B**). Thus the 0-ms-gap stimuli contained small amplitude reductions in both the WF and BF conditions. For each FC, the stimulus presentation and other parameters were the same as in Experiment 1.

### DATA ACQUISITION

MEG measurement was conducted in the Brain Center in Kyushu University Hospital. AEFs were measured using a whole-head 306-channel biomagnetometer system (Elekta, Neuromag, Helsinki, Finland) in a quiet, magnetically shielded room. The detector array comprised 102 identical triple-sensor elements, with each sensor element comprising two orthogonally oriented planar-type gradiometers and one magnetometer. Before recording, four head-position indicator (HPI) coils were attached to the scalp, and a 3D digitizer was used to measure head shapes with respect to the HPI coils. Magnetic responses were digitally sampled at 1000 Hz, and online filtered with a bandpass of 0.1–330 Hz. MRI data were acquired using a 3.0-T high resolution MRI scanner (Achieve, Philips N.V. Eindhoven, The Netherlands) for analysis (TE, 60 ms; TR, 100 ms; voxel size, 1.5mm × 1.5mm × 1.5mm) and interpretation of MEG data.

### SIGNAL PROCESSING AND SOURCE RECONSTRUCTION

After recording, Maxfilter (Taulu et al., 2005) was used to reduce artifact signals arising from outside the sensor array. A 1–100 Hz off-line bandpass filter and a 60 Hz notch filter were applied to highlight the AEFs. AEFs measured from ∼80 responses for each FC were averaged for each gap duration. Using the averaged data, we focused on the contralateral hemisphere because AEFs are usually larger there than they are ipsilaterally (Pantev et al., 1986). The peak latencies and amplitudes of the AEFs were picked up from the gradiometer that showed the most salient activation in the AEFs for each FC.

Following off-line signal processing, we performed an MEG source reconstruction. A distributed source model of the MEG signals (recorded from the entire head surface) was estimated using MNE to obtain the current strength of cortical sources. This method offers high spatial resolution for detecting simultaneous magnetic sources distributed across the entire cortical surface. The precise procedure for performing MNE has been described elsewhere (Hamalainen and Ilmoniemi, 1994; Molins et al., 2008). Each participant’s cortical surface was reconstructed from high-resolution T1-weighted MR images using FreeSurfer software (Fischl et al., 1999). An anatomical MRI image was co-registered with the MEG head coordinate system using head-shape points obtained by Polhemus measurement.

An inverse solution was calculated based on the forward solution that models the signal pattern generated by a unit dipole at each location on the cortical surface using a single homogeneous realistic head model and a boundary element method (BEM). The activation at each cortical location was estimated at each time point of the activity, and was simultaneously estimated using a noise-normalized linear estimation approach [dynamic statistical parametric maps (dSPM); Dale et al., 2000]. A noise covariance matrix was created using pre-trigger periods from -100 to 0 ms via trigger onset. The activation patterns derived from the analysis were mapped onto the cortical surface images of each participant to make visualization clear. Each participant’s data were transformed into a standard brain (MNI305; Collins et al., 1994) to estimate the source activations across subjects on the same scale (Fischl et al., 1999).

### GROUP ANALYSIS

**FIGURE 2:**
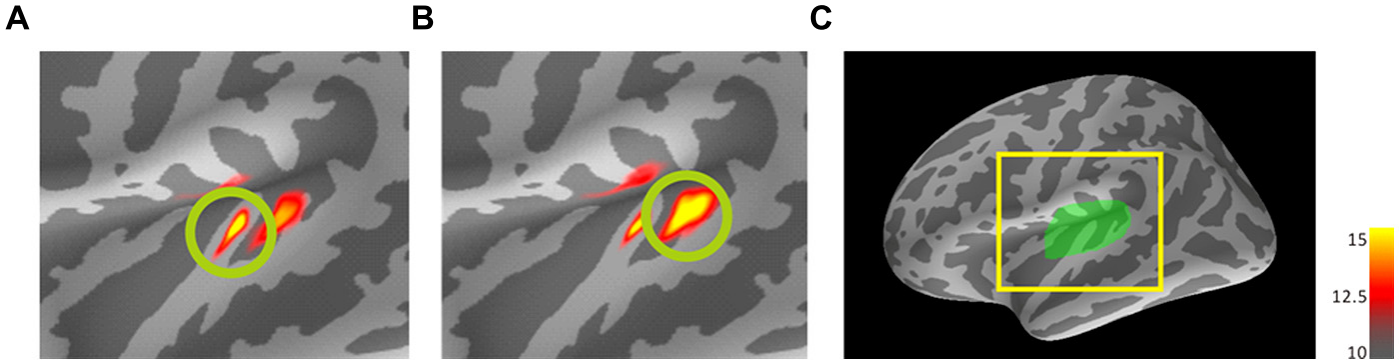
**Dynamic statistical parametric maps (dSPM) results of mean activations to the trailing marker on the standard brain for 800-Hz **(A)** and 3200-Hz **(B)** within-frequency (WF) trailing markers.** Source activation of AEF responses in auditory cortex ROI of the left hemisphere **(C)** to the trailing marker both for 30- and 80-ms gap durations were transformed from individual brains into the standard brain (MNI305) and averaged across 10 participants with standardization. The coloring threshold levels were set at fthres (low threshold) = 10, fmid (middle) = 12.5, and fmax (maximum) = 15 for all figures. **(C)** A lateral view of the left hemisphere of the standard brain showing the region of interest (left Heschl’s gyrus). **(A)** and **(B)** represent enlargements of the area surrounded by the yellow square. dSPM, dynamic statistics parameter mapping. *MNI*, Montreal Neurological Institute.

**FIGURE 3:**
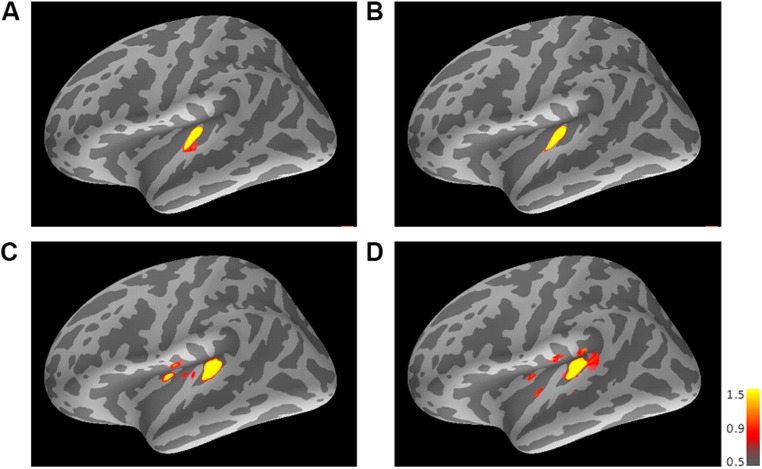
**Mean activations in response to the trailing marker depicted on the standard brain for each condition: **(A)** 800/800, **(B)** 3200/800, **(C)** 3200/3200, and **(D)** 800/3200 in Experiment 1.** Source activation results of the averaged AEF responses to the trailing marker were obtained after transferring data from marked individual ROIs to the standard brain (MNI305). The threshold levels were set at fthres=5, fmid=9, and fmax=15 for all figures. MNI, Montreal Neurological Institute.

**FIGURE 4:**
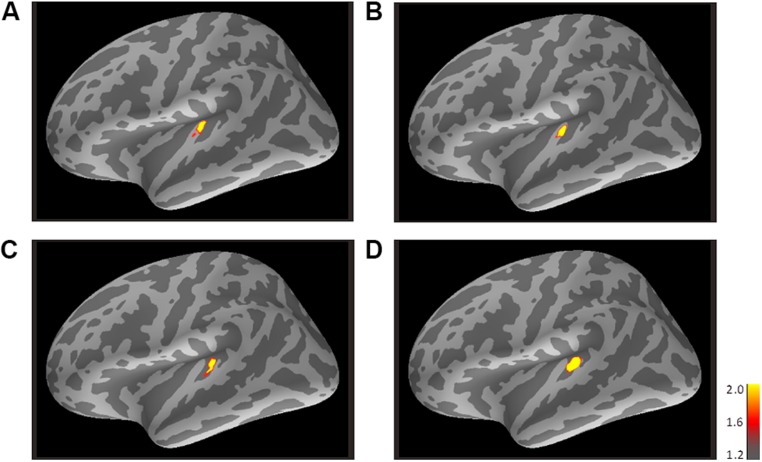
**Mean activations in response to the trailing marker depicted on the standard brain for each condition: **(A)** 800/800, **(B)** 3200/800, **(C)** 3200/3200, and **(D)** 800/3200 in Experiment 2.** Source activation results of the averaged AEF responses to the trailing marker were obtained after transferring data from marked individual ROIs to the standard brain (MNI305). The threshold levels were set at fthres=1.2, fmid=1.6, and fmax=2.0 for all figures. MNI, Montreal Neurological Institute.

To confirm the primary activated areas in each of the four frequency conditions (800/800, 3200/3200, 3200/800, and 800/3200), activation maps at the peak latencies (N1m) of the trailing markers were estimated using dSPM and averaged with standardization (divided by max value) after transforming them into the standard brain. We estimated the target areas in each of the four frequency conditions in two steps. First, we averaged the activation map using a set ROI that covered the transverse temporal gyrus and its immediate vicinity (i.e., the auditory cortex; Pantev et al., 1988) to obtain a common activated area across all participants (**Figure 2**). Second, referencing the common activated area marked by the first step and the strongest activation in the auditory cortex from each individual, we marked the iROIs on the auditory cortex in the left hemisphere of each participant’s cortex for all four conditions. Then, activity of each marked iROI was re-transformed into the standard brain and averaged again (**Figures 3** and **4**). After obtaining the iROIs corresponding to the 800- and 3200-Hz trailing markers, each activity pattern and tendency was examined individually. To statistically evaluate the accuracy of source localization, the center locations of N1m responses to the trailing markers were estimated for all four FCs in each participant. The center locations for the marked 800- and 3200-Hz iROIs were calculated and transformed into the standard brain so that location estimates would be on the same scale. Finally, a center location on the standard brain was estimated for each FC using weighted averaging that followed our established methods (Hironaga et al., 2014). The coordinate system used to express the location is based on the MNI Talairach. The x-axis indicates the medial/lateral direction, y-axis indicates the anterior/posterior direction, and z-axis indicates the inferior/superior direction. The RA in each iROI for each stimulus was extracted from each individual, and the activities were also averaged with standardization. The N1m peak latencies for both the leading and trailing markers were extracted from RAs for all conditions and corresponding amplitudes were evaluated. We defined the peak latencies of the 0-ms gap in the WF condition in Experiment 2 as a peak that occurred within the 100–200-ms time window after the onset of the trailing marker (i.e., gap offset).

## RESULTS

### SOURCE ACTIVATION GROUP ANALYSIS

#### Experiment 1

**Figure 3** shows the averaged AEF responses of 10 participants to the trailing marker after converting the activity in marked individual iROIs to the standard brain. The areas showing responses to the 800-Hz tone were located in anterior Heschl’s gyrus (HG), while those to the 3200-Hz tone were located in posterior HG. Responses to the 800-Hz tone appeared concentrated in a single area regardless of condition (**Figures 3A,B**), while those to the 3200-Hz tone were dispersed across the auditory cortex (**Figures 3C,D**). This was especially true for the 800/3200 condition (**Figure 3D**). **Table 1** gives the mean estimated centers of N1m responses to the leading marker for both frequencies, and **Table 2** shows those to the trailing marker for all four conditions.

**Table 1:** The center location of leading markers’ N1m on the standard brain in Experiments 1 and 2.

	MNI coordinates*	800 Hz	3200 Hz
Experiment 1	*x*	-43.5 (±2.4)	-47.3 (±6.2)
	*y*	-25.0 (±2.3)	-30.6 (±6.5)
	*z*	8.0 (±1.3)	8.5 (±4.2)
Experiment 2	*x*	-43.7 (±3.3)	-47.0 (±5.3)
	*y*	-25.6 (±2.5)	-30.8 (±3.0)
	*z*	7.6 (±1.9)	4.9 (±2.7)

Coordinates are given as mean (±SD).

*Montreal Neurological Institute (MNI) coordinates [Right Anterior Superior (RAS) coordinate in the standard brain].

**Table 2:** The center location of trailing markers’ N1m on the standard brain for each frequency combination in Experiments 1 and 2.

	MNI coordinates*	800/800	3200/3200	800/3200	3200/800
Experiment 1	*x*	-44.6 (±3.1)	-47.9 (±6.4)	-44.7 (±5.6)	-44.4 (±2.4)
	*y*	-24.1 (±3.0)	-30.0 (±6.2)	-27.2 (±7.0)	-23.9 (±2.5)
	*z*	7.4 (±1.5)	6.7 (±4.0)	7.1 (±4.4)	7.3 (±1.5)
Experiment 2	*x*	-43.0 (±3.9)	-48.4 (±6.7)	-45.6 (±3.0)	-44.4 (±2.5)
	*y*	-26.0 (±3.8)	-28.8 (±3.8)	-30.5 (±1.9)	-24.9 (±3.6)
	*z*	8.0 (±2.2)	4.7 (±3.1)	5.2 (±2.3)	7.3 (±1.5)

Coordinates are given as mean (±SD).

*Montreal Neurological Institute (MNI) coordinates [Right Anterior Superior brain (RAS) coordinate in the standard brain].

An ANOVA was performed using IBM SPSS statistics 21 (IBM Co. Ltd., Armonk, NY, USA) to assess the center locations of N1m iROI, as well as the amplitudes and the latencies of RA patterns for each condition. To check whether the N1m sources for the leading and trailing markers were localized, we chose “frequency” as a factor for both leading and trailing marker. For the frequency factor of the leading marker, we averaged the coordinates of the 800/800 and 800/3200 conditions and those of the 3200/3200 and 3200/800 conditions. In contrast, for the frequency factor of the trailing marker, we averaged the coordinates of the 800/800 and 3200/800 conditions and those of the 3200/3200 and 800/3200 conditions. One-way (Frequency: 800, 3200) ANOVAs were performed separately on the center coordinate values of the three axes (x, y, and z) obtained for the leading and the trailing markers. The Greenhouse–Geisser correction was applied when the assumption of sphericity was violated in the dependent measures. *Post hoc* Bonferroni corrections multiple comparisons were applied when required. The $\eta_{p}^{2}$ (partial eta-squares) were calculated for the quantitative comparison of effect sizes. For the leading marker, the main effect of frequency was significant in the y-axis [*F*_(1,9)_ = 7.02, *p*<0.05, $\eta_{p}^{2}$ = 0.48], but not in the x-axis [*F*_(1,9)_ = 3.16, *p*=n.s., $\eta_{p}^{2}$ = 0.26] or the z-axis [*F*_(1,9)_ = 0.12, *p*=n.s., $\eta_{p}^{2}$ = 0.01]. The center location of the 800-Hz N1m (*y*=-24.99) was more anterior than that of the 3200-Hz N1m (*y*=-30.62). For the trailing marker, the main effect of trailing frequency was significant in the y-axis [*F*_(1,19)_ = 8.89, *p*<0.01, $\eta_{p}^{2}$ = 0.32], but not in the x-axis [*F*_(1,19)_ = 1.46, *p*=n.s., $\eta_{p}^{2}$ = 0.07] or the z-axis [*F*_(1,19)_ = 0.17, *p*=n.s., $\eta_{p}^{2}$ = 0.01]. The center of the 800-Hz N1m (*y*=-23.96) was more anterior than that of the 3200-Hz N1m (*y*=-28.63).

#### Experiment 2

**Figure 4** shows the averaged AEF responses of 6 participants to the trailing marker in Experiment 2. Core activations appeared in almost identical locations to those obtained in Experiment 1 (**Figure 3**), but dispersion of the individual iROIs was much less in the 3200-Hz condition (**Figures 4C,D**). The mean estimated centers of N1m responses to the leading and trailing markers are given in **Tables 1** and **2**. As in Experiment 1, one-way (Frequency: 800, 3200) ANOVAs were performed separately for the leading and trailing markers on each coordinate axis. For the leading marker, the main effect of frequency was significant in all axes [x-axis: *F*_(1,5)_ = 9.38, *p*<0.05, $\eta_{p}^{2}$ = 0.65; y-axis: *F*_(1,5)_ = 16.31, *p*<0.01, $\eta_{p}^{2}$ = 0.77; z-axis: *F*_(1,5)_ = 8.87, *p*<0.05, $\eta_{p}^{2}$ = 0.64]. The center of the 800-Hz N1m was more lateral (*x*=-46.42), anterior (*y*=-25.64), and superior (*z*=8.01) than that of the 3200-Hz N1m (*x*=-47.00, *y*=-30.80, *z*=5.95). For the trailing marker, the main effect of frequency was also significant for all axes [x-axis: *F*_(1,11)_ = 6.11, *p*<0.05, $\eta_{p}^{2}$ = 0.36; y-axis: *F*_(1,11)_ = 24.20, *p*<0.001, $\eta_{p}^{2}$ = 0.69; z-axis: *F*_(1,11)_ = 17.91, *p*<0.091, $\eta_{p}^{2}$ = 0.62]. The center of the 800-Hz N1m for the trailing marker was more lateral (*x*=-43.68), anterior (*y*=-25.46), and superior (*z*=7.64) than that of the 3200-Hz N1m (*x*=-47.00, *y*=-29.60, *z*=4.94).

### ANALYSIS OF REGIONAL ACTIVITY

#### Experiment 1

**FIGURE 5:**
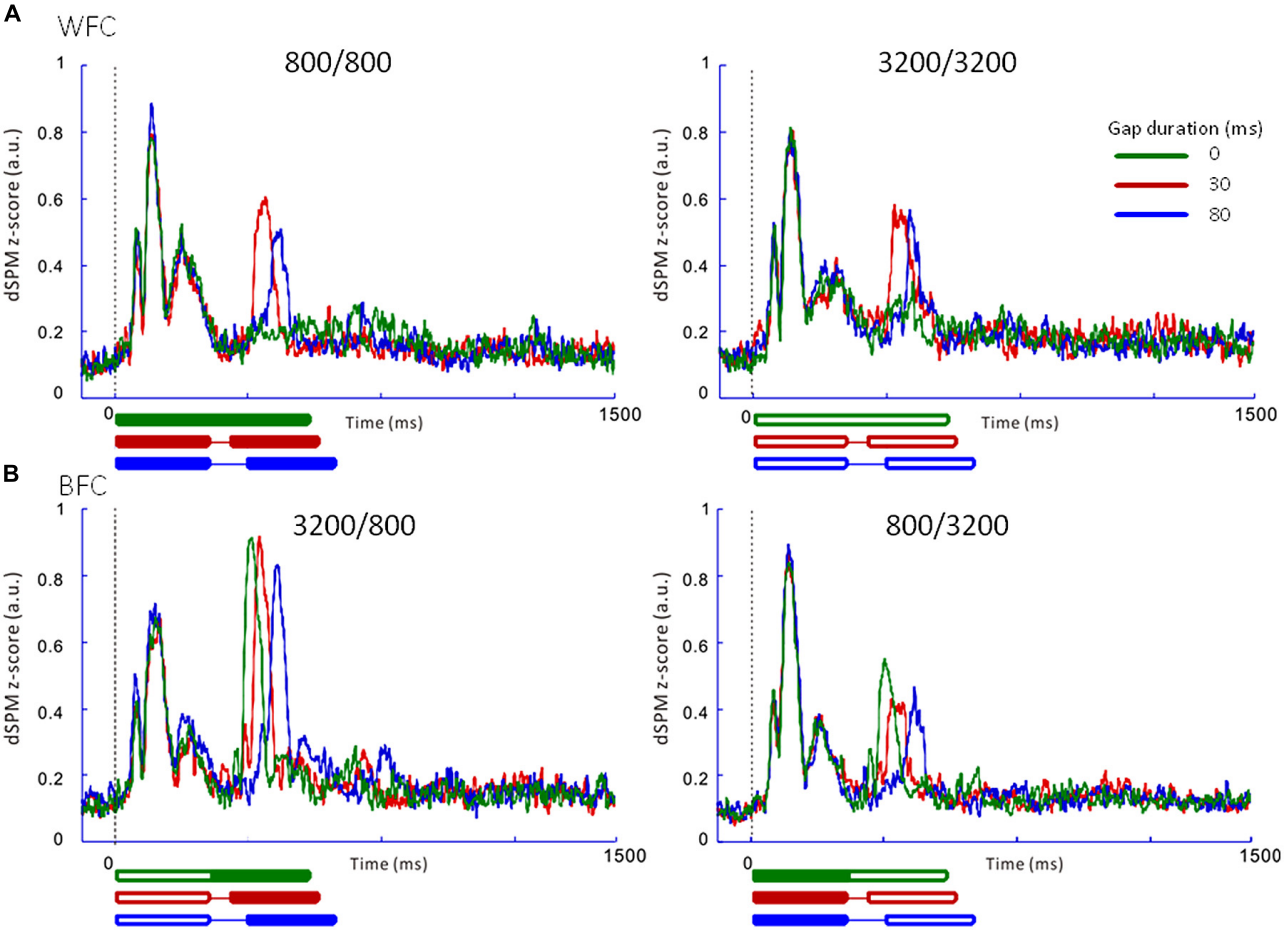
**Averaged regional activities (RAs) in the left auditory inspection region of interest from 10 participants in Experiment 1. (A)** RAs for the WF conditions. **(B)** RAs for the BF conditions. Thick lines under the horizontal axis of each RA represent the time range of stimulus presentation for the 0-ms (*green*), 30-ms (*red*), and 80-ms (*blue*) gaps. Filled lines denote the 800-Hz markers, while open lines denote the 3200-Hz markers.

**Figure 5** presents the averaged RAs for the trailing markers from 10 participants that were extracted from individually marked iROIs. While onset responses for the trailing marker were not observed for the 0-ms gap in the WF condition (**Figure 5A**, *green line*), they were clearly evident in the BF condition (**Figure 5B**, *green line*). We also compared the RAs from **Figure 5** with sensor-level average waveforms (data not shown) and confirmed that the N1m in our study was equivalent to a P1-N1-P2 response pattern (e.g., Ross et al., 2010). For RA amplitudes, we used the relative amplitudes (peak value of the trailing marker divided by that of the leading marker) as an independent variable of interest (**Table 3**). The values for individual participants were subjected to a 2 [Frequency (Fr): 800 vs. 3200 Hz] × 2 [Gap duration (GD): 30 vs. 80 ms] ANOVA for the WF condition and a 2 (Fr: 800 vs. 3200 Hz) × 3 (GD: 0, 30, 80 ms) ANOVA for the BF condition. For the WF condition, we observed a significant main effect of GD [*F*_(1,9)_ = 5.82, *p*<0.05, $\eta_{p}^{2}$ = 0.39], but no significant main effect of Fr [*F*_(1,9)_ = 0.02, n.s., $\eta_{p}^{2}$ = 0.002]. The peak amplitudes for 30-ms trailing marker were larger than those for the 80-ms trailing marker. In the BF condition, ANOVA revealed a significant main effect of Fr [*F*_(1,9)_ = 27.02, *p*<0.001, $\eta_{p}^{2}$ = 0.75]. The peak amplitudes for the 800-Hz trailing marker were larger than those for the 3200-Hz trailing marker. There was no significant main effect of GD [*F*_(2,18)_ = 2.17, n.s., $\eta_{p}^{2}$ = 0.19]. For both WF and BF conditions, the interaction between Fr and GD was not significant [WF: *F*_(1,9)_ = 3.39, n.s., BF: *F*_(2,8)_ = 2.56, n.s.].

**Table 3:** Relative amplitudes of the regional activities in the iROI in Experiments 1 and 2.

	Gap	Trailing/Leading markers			
		800/800	3200/3200	800/3200	3200/800
Experiment 1	0 ms	–	–	0.71 (±0.27)	1.22 (±0.27)
	30 ms	0.93 (±0.52)	0.80 (±0.29)	0.58 (±0.24)	1.41 (±0.49)
	80 ms	0.66 (±0.26)	0.78 (±0.31)	0.54 (±0.19)	1.16 (±0.32)
Experiment 2	0 ms	0.43 (±0.22)	0.48 (±0.27)	0.57 (±0.21)	1.11 (±0.56)
	30 ms	0.68 (±0.28)	0.60 (±0.40)	0.45 (±0.12)	0.91 (±0.26)
	80 ms	0.59 (±0.21)	0.63 (±0.35)	0.47 (±0.16)	0.86 (±0.18)

*Amplitudes are given as mean (±SD).*

**Table 4** shows the peak latencies of the RAs from the iROI obtained in Experiment 1. For the leading marker, we performed a 4 (FC: 800/800, 800/3200, 3200/800, 3200/3200) × 3 (GD: 0, 30, 80 ms) ANOVA. For the trailing marker, we performed a 2 (Fr: 800 vs. 3200 Hz) × 2 (GD: 30 vs. 80 ms) ANOVA for the WF condition and a 2 (Fr: 800 vs. 3200 Hz) × 3 (GD: 0, 30, 80 ms) ANOVA for the BF condition. For the leading marker, ANOVA showed no significant main effect of FC [*F*_(3,27)_ = 0.99, n.s., $\eta_{p}^{2}$ = 0.10] or GD [*F*_(2,18)_ = 0.54, n.s., $\eta_{p}^{2}$ = 0.06]. Peak RA latencies in response to the leading marker appeared to be around 110 ms after stimulus onset for all FCs, which corresponded to the N1m in the sensor-level AEF. For the trailing marker, there was a significant main effect of GD in both conditions [WF: *F*_(1,9)_ = 406.44, *p*<0.001, $\eta_{p}^{2}$ = 0.98; BF: *F*_(2,18)_ = 344.55, *p*<0.001, $\eta_{p}^{2}$ = 0.98], but no significant main effect of Fr in either condition [WF: *F*_(1,9)_ = 0.00, n.s., $\eta_{p}^{2}$ = 0.00; BF: *F*_(1,9)_ = 1.25, n.s., $\eta_{p}^{2}$ = 0.12]. Similar to the leading marker, peak RA latencies in response to the trailing marker appeared to be around 110 ms after stimulus onset. For example, in the 800/3200-BF case, the average onset latencies for trailing markers were 108, 138, and 194 ms for the 0-, 30-, and 80-ms gaps, respectively. The differences of these latencies (30 ms between 0- and 30-ms gaps and 55 ms between 30- and 80-ms gaps) corresponded to the differences in gap durations.

**Table 4:** Latencies (ms) of the regional activities in the iROI in Experiments 1 and 2.

		Frequency combination							
		800/800		3200/3200		800/3200		3200/800	
	Gap	Leading marker	Trailing marker	Leading marker	Trailing marker	Leading marker	Trailing marker	Leading marker	Trailing marker
Experiment 1	0 ms	115.5 (±12.9)	–	111.7 (±11.3)	–	112.8 (±10.5)	107.8 (±14.3)	116.7 (±13.5)	106.7 (±6.8)
	30 ms	110.7 (±7.2)	142.9 (±13.3)	115.8 (±13.8)	144.7 (±11.3)	115.1 (±12.1)	138.0 (±16.6)	117.0 (±14.7)	132.3 (±2.8)
	80 ms	109.9 (±6.7)	189.8 (±9.9)	110.9 (±9.8)	188.1 (±19.0)	113.2 (±11.1)	194.4 (±11.0)	117.1 (±17.1)	194.4 (±11.0)
Experiment 2	0 ms	101.5 (±3.6)	137.3 (±16.4)	103.8 (±6.2)	149.8 (±19.7)	104. 0 (±9.9)	103.8 (±6.5)	102. 3 (±4.2)	106.2 (±11.3)
	30 ms	102.2 (±4.4)	133.5 (±5.5)	106.3 (±5.0)	137.0 (±9.6)	107.0 (±8.9)	138.7 (±7.8)	101.2 (±6.0)	139.0 (±5.4)
	80 ms	104.2 (±4.7)	186.0 (±9.5)	103.3 (±2.9)	178.3 (±14.1)	103.0 (±8.2)	192.0 (±11.9)	105.8 (±6.7)	192.0 (±12.1)

*Latencies are given as mean (±SD).*

#### Experiment 2

**FIGURE 6:**
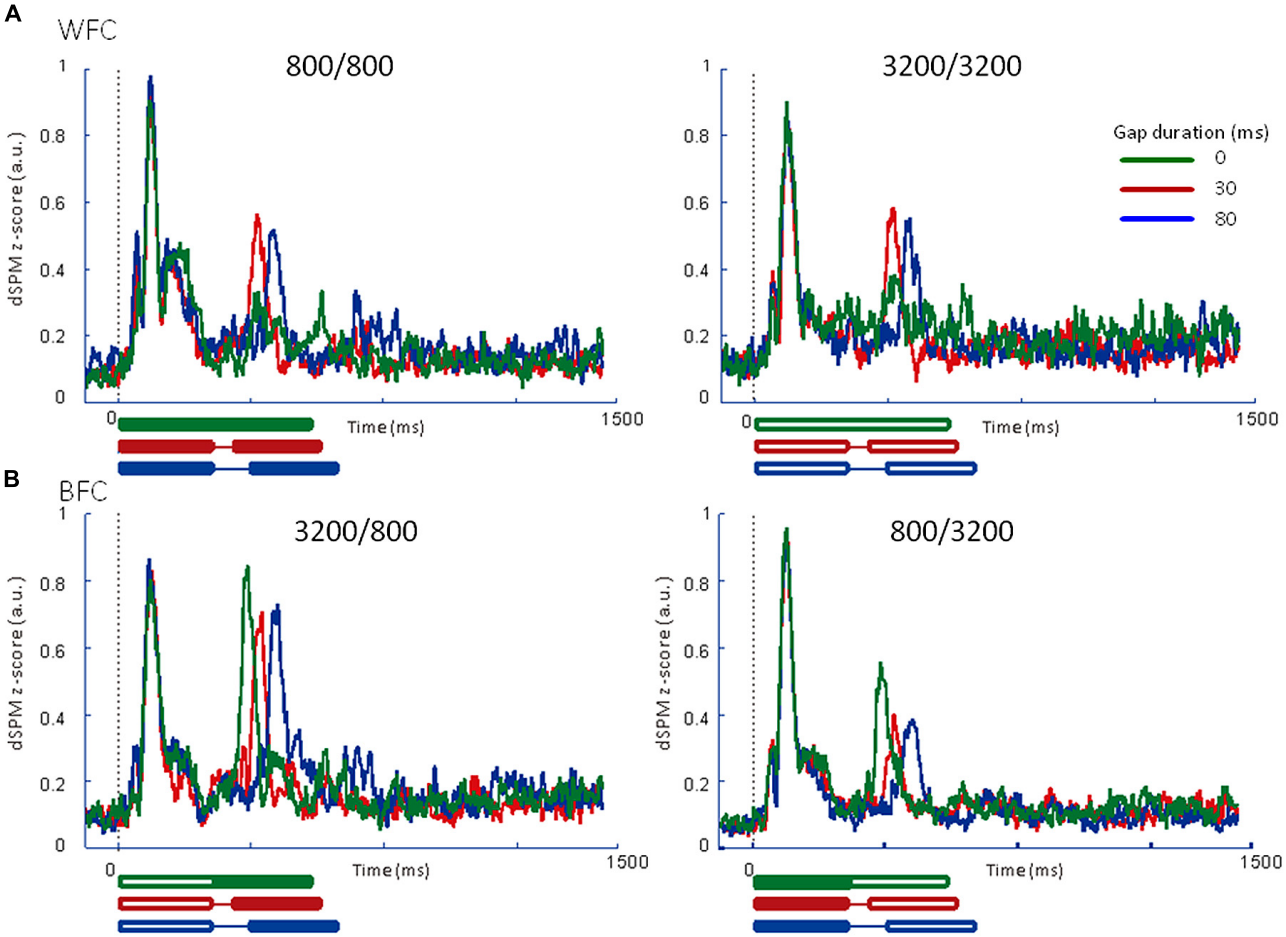
**Averaged regional activities (RAs) in the left auditory inspection region of interest from 6 participants in Experiment 2. (A)** RAs for the WF conditions. **(B)** RAs for the BF conditions. Thick lines under the horizontal axis of each RA represent the time range of stimulus presentation for the 0-ms (*green*), 30-ms (*red*), and 80-ms (*blue*) gaps. Filled lines denote the 800-Hz markers, while open lines denote the 3200-Hz markers.

We calculated the averaged RAs for the trailing markers (**Figure 6**) and the relative amplitudes of the RAs in the iROI (**Table 3**). In the WF condition, onset responses for the trailing marker were observed for the 0-ms gap condition (**Figure 6A**, *green line*), but amplitudes were smaller than those of the 30-ms and 80-ms gap conditions (**Figure 6A**, *red* and *blue* lines). In the BF condition, the onset responses for the trailing marker were observed for the 0-ms gap condition (**Figure 6B**, *green line*). This tendency is consistent with the results in Experiment 1. We performed a 4 (FC: 800/800, 800/3200, 3200/800, 3200/3200) × 3 (GD: 0, 30, 80 ms) ANOVA on the peak amplitude for the trailing marker. The result showed a significant main effect of FC [*F*_(3,15)_ = 9.93, *p*<0.01, $\eta_{p}^{2}$ = 0.67] but not for GD [*F*_(2,10)_ = 0.63, n.s.]. The peak amplitudes for the 3200/800 trailing marker were larger than those for the 800/3200 trailing marker (*t*=0.57, *p*<0.05). The interaction between FC and GD was not significant [*F*_(1.32,6.60)_ = 0.20, n.s.].

**Table 4** shows the peak RA latencies in the iROI from Experiment 2. A 4 (FC: 800/800, 800/3200, 3200/800, 3200/3200) × 3 (GD: 0, 30, 80 ms) ANOVA was performed on the peak RA latencies of the leading marker as well as the trailing marker to observe the timing of the onset responses for both markers. For the leading marker, no significant main effect of FC [*F*_(3,15)_ = 0.356, n.s., $\eta_{p}^{2}$ = 0.67] or GD [*F*_(2,10)_ = 1.57, n.s., $\eta_{p}^{2}$ = 0.24] were found. Peak RA latencies in response to the leading marker appeared to be around 100 ms after stimulus onset for all FCs. For the trailing marker, ANOVA revealed a significant main effect of GD [*F*_(2,10)_ = 204.50, *p*<0.001, $\eta_{p}^{2}$ = 0.98], but not for FC [*F*_(3,15)_ = 2.17, n.s., $\eta_{p}^{2}$ = 0.35]. The interaction between FC and GD was significant [*F*_(2.74,13.68)_ = 16.86, *p*<0.01, $\eta_{p}^{2}$ = 0.76]. In WF conditions, there was no difference in latency between 0- and 30-ms gaps (800/800: 0 vs. 30 ms: *t*=3.83; 3200/3200: 0 vs. 30 ms: *t*=12.83). The N1m peak latency for the 0-ms gap in WF conditions appeared to be around 30 ms after the onset of the trailing marker.

## DISCUSSION

This study investigated spatio-temporal characteristics of cortical responses corresponding to WF and BF gap detection in human auditory cortex using MEG. In terms of their temporal characteristics, in Experiment 1 we found that N1m amplitude for the trailing marker in WF condition was larger for 30-ms gaps than for 80-ms ones, while in BF condition, it was larger when the training marker was 800 Hz than when it was 3200 Hz. In Experiment 2, N1m amplitude was larger for 800-Hz markers than for 3200-Hz markers, regardless of the type of condition. Spatially, Experiment 1 showed that 800 and 3200 Hz markers generated activation that differed in the anterior-posterior direction, while in Experiment 2 activity differed in all directions. These results indicate different activation patterns for WF and BF conditions in spatial and temporal dimensions.

### AEF SOURCE LOCALIZATION DURING WF AND BF CONDITIONS

The MNE results from the group analysis, which focused on an onset response to the trailing marker, were in line with previous MEG and fMRI studies. Our current results show that activations were estimated to be in the auditory cortex in both Experiments 1 and 2: 800-Hz responses are located more anteriorly than 3200-Hz ones (**Figure 6B**). Other MEG studies have shown that when stimulus frequencies are increased, the N1m shifts to lateral to medial direction along the surface of the auditory cortex (Romani et al., 1982; Pantev et al., 1988, 1995). Several fMRI studies have reported that areas most responsive to high frequency tones are located in the posterior and medial regions, while those selective for low frequency tones are located at the anterior and lateral regions (Talavage et al., 2000; Formisano et al., 2003).

The source locations activated by the 3200-Hz tone were less concentrated, while those activated by the 800-Hz tone were reproducible and stable (especially in Experiment 1), as indicated by the relatively larger standard deviations in the y-axis for 3200-Hz tones compared with 800-Hz tones (**Figure 3** and **Table 2**). Additionally, the statistical significance of differences along the x- and z-directions differed between the two experiments. Because the participants of Experiments 1 and 2 were not identical, differences in the estimated center locations between the two experiments might in part be owing to differences in auditory cortex anatomy across individuals. Indeed, inter-participant variability in the location of the recorded cortical activity has often been reported in MEG and fMRI studies (e.g., Formisano et al., 2003; Lütkenhöner et al., 2003; Zatorre and Schönwiesner, 2011).

### REGIONAL ACTIVITY FOR THE WF AND BF CONDITIONS

In both Experiments 1 and 2, in the WF condition, stable activation patterns of the N1m-peak amplitude were observed for both FCs (800/800 and 3200/3200) across 30- and 80-ms gap durations. In contrast, in the BF condition, the RA pattern for the trailing marker was different depending on the trailing markers’ frequency (3200/800 or 800/3200; **Figures 5B** and **6B**). In both Experiments 1 and 2, amplitudes were significantly higher for 800-Hz tones than for 3200-Hz ones.

The results of Experiment 2 showed onset responses to the trailing marker in all conditions including WF with a 0-ms gap. Two of the six participants exhibited onset responses to the trailing marker with a 0-ms gap. When re-analyzing the N1m response in the WF condition after excluding these two participants, the N1m amplitudes to the 0-ms gap condition (0.29 for 800 Hz and 0.31 for 3200 Hz) were as small as waveform baseline (about 0.2, as indicated in **Figures 5** and **6**), although there were no significant differences among the three gap durations [*F*_(2,6)_ = 3.34, n.s., $\eta_{p}^{2}$ = 0.53]. In the WF condition, neurophysiological sensory sensitivity to the gap might be highly correlated with its psychophysical threshold. Indeed, amplitude of gap-evoked responses has been shown to increase as a function of gap duration, and be correlated with the psychological threshold of each participant (Witton et al., 2012). Therefore, we assume that differences in N1m amplitude for the 0-ms gap in the WF condition might be related to individual differences in the sensitivity to gaps. For the BF condition, onset responses clearly appeared for all trailing markers, even when the gap was lacking. The response to the 0-ms gap in the BF condition was close in amplitude to those in which the gap lasted 30 or 80 ms, making comparisons between the gap and no-gap responses difficult for the BF condition. These complexities of response patterns in the BF condition might be connected to the large individual differences that are seen in gap-detection thresholds during the BF condition (Formby and Forrest, 1991).

The difference between WF and BF conditions in onset response to trailing marker when no gap was present might indicate a difference in the underlying neural processing for WF- and BF-gap detection. For the WF condition, responses to the onset of the leading and trailing markers occurred for a single frequency in temporally close timing. In this case, a neuronal population in a single area should activate to respond to the leading and the trailing marker. As there was no additional cue indicating frequency change after the gap, the response to the trailing marker was not robust, especially when the amplitude difference between the two markers was absent or very small (i.e., a 0-ms gap). For the BF condition, the different responses to the onset of the leading and trailing markers occurred for different frequencies. Because the response to the trailing marker occurred in neural populations in different areas than the leading marker, the onset response to the trailing markers should be salient even when a gap is absent (Phillips, 1999; Eggermont, 2000; Heinrich et al., 2004; Lister et al., 2007).

### FUNCTIONAL CHANNELS AND TONOTOPIC ORGANIZATION

In WF-gap detection, both leading and trailing markers are considered to be processed in a frequency-selective auditory pathway (i.e., channel) in the auditory stream for detecting temporal discontinuity, and WF-gap detection can be achieved peripherally with relative ease, with a gap-detection threshold around 2–3 ms (Plomp, 1964; Penner, 1977). Such small gap-detection thresholds have been explained in terms of the properties of the auditory periphery (Shailer and Moore, 1983). Conversely, in BF-gap detection, the leading and trailing markers are processed through separate frequency pathways because both markers usually have different or non-overlapping spectral content. BF-gap detection is presumably performed centrally (Phillips et al., 1997; Phillips, 1999; Eggermont, 2000). Multi-unit recordings in cat primary auditory cortex showed that the firing patterns of neurons in auditory cortex reflect minimum detectable gap thresholds that are similar to thresholds measured psychophysically in humans (Phillips et al., 1997; Eggermont, 2000). Eggermont (2000) suggested that the secondary auditory cortex and anterior auditory field are also involved in gap detection. Because the N1m response to the sound marker was suggested to be related to the psychophysical threshold in humans (Witton et al., 2012), the N1m sources, such as the supra temporal plane, could be involved in gap detection as well. In humans, tonotopic organization in auditory cortex has been verified with MEG (Romani et al., 1982; Pantev et al., 1988), EEG (Bertrand et al., 1988), and fMRI (Talavage et al., 2000; Formisano et al., 2003). Tonotopic organization has been observed in the superior temporal plane, including HG, Heschl’s sulcus, and the superior temporal gyrus (e.g., Talavage et al., 2000). Examining the frequency channel from the perspective of tonotopic alignment in human auditory cortex could yield new and interesting findings.

So far, studies have reported modulation of EEG components related to the processing of the leading and the trailing markers via a sensor-level approach (Heinrich et al., 2004; Lister et al., 2007). Compared with EEG, MEG measurement allows for more advanced analyses, especially in respect to the spatial resolution. By employing MEG, we showed the spatial separation between the frequency channels corresponding to the leading and trailing markers in terms of tonotopic organization in the auditory cortex. We assumed that frequency channels can be represented by iROI and RAs in iROI (i.e., RAs; **Figures 2**–**6**). The investigation of iROI and RA in the auditory cortex is the first step to delineate cortical activation related to the processing of gap detection. Our approach using iROI and RA will be useful for investigating the gap-detection mechanism.

### LIMITATIONS AND FUTURE RESEARCH

Using MEG/EEG for source localization of auditory responses to high frequency ranges can be difficult because of their limited spatial resolution. Studies that record auditory evoked brain responses often adopt 500–2000 Hz tones because the sources for these frequency tones have been consistently estimated to be in the auditory cortex (e.g., Stapells et al., 1994). Because we used a higher frequency tone (i.e., 3200 Hz) than usually examined frequency ranges, the results of iROI did not exhibit concentrated locations. Therefore, we were unable to make systematic analyses across the participants, i.e., we were not able to mark ROI on the standard brain first and then project it onto the individual’s brain. We need to accumulate more evidence regarding the tonotopic organization of wider frequency ranges to confirm the reliability of our results. In addition, the gap duration adopted in our current study was determined somewhat arbitrarily and we did not measure gap-detection thresholds to WF and BF stimuli individually for each participant. Therefore, whether the durations used in our experiments really reflect the gap thresholds of the participants is unclear. Moreover, we did not measure the hearing levels for each participant, and we are unable to say whether auditory sensitivity to the tones might contribute to the amplitude differences found in the current data. A more detailed analysis will require several patterns of FCs for BF stimuli and individual gap-detection thresholds for each participant under appropriate stimulus settings. Our RA analysis that was based on tonotopic organization has provided a clue that helps us understand how gap detection in the auditory cortex is accomplished.

## CONCLUSION

Auditory gap detection is one of the most popular issues with respect to human mental chronometry. Here, we used MEG and focused on how the auditory cortex responds to gaps bounded by tones of either the same or different frequencies. The source-activation maps and regional time-course waveforms indicated distinct patterns between the WF and BF conditions at the cortical level. One clear difference in temporal patterns between the two conditions was in the sensitivity to trailing marker onsets when no gap was present: the onset responses to the trailing marker depended on length of the gap in the WF condition, whereas it depended mainly on the differences in tonal frequency in the BF condition. Further, we showed frequency sensitive brain activity in the human auditory cortex that was related to gap detection and based on tonotopic organization. Frequency channels can be represented by iROI and RAs in iROI (i.e., RA). Although future studies are required, our findings open a new door to better understanding of gap-detection processing.

## Conflict of Interest Statement

The authors declare that the research was conducted in the absence of any commercial or financial relationships that could be construed as a potential conflict of interest.
